# Infectious adverse effects during treatment with IL-1 inhibitors in patients with systemic juvenile idiopathic arthritis and autoinflammatory diseases

**DOI:** 10.1186/1546-0096-12-S1-P134

**Published:** 2014-09-17

**Authors:** Laura Fernandez-Silveira, M Isabel González-Fernández, Berta López-Montesinos, Silvia Benito-Costey, Inmaculada Calvo-Penadés

**Affiliations:** 1 Pediatric Reumatology, Hospital la Fe, Valencia, Spain

## Introduction

IL-1 plays an important role in the pathogenesis of both illnesses. The anti-IL1 therapy (anakinra/ canakinumab) had been proved to be very effective in their treatment. The infections related to these treatments in children had been hardly described in literature.

## Objectives

To describe the infectious complications in children with systemic juvenile idiopathic arthritis (SJIA) or autoinflammatory diseases (AD) while receiving anti-IL1 drugs.

## Methods

All the patients who had received IL1 inhibitors from January 2005 to January 2014 in our unit were included. We recorded the length of the exposition to anakinra or canakinumab, diagnosis, previous exposition to a different biologic treatment and moderate or severe infections.

The patients with SJIA were defined by the ILAR criteria. In the AD group were included familiar mediterranean fever (FMF), Hiper IgD syndrome (HIDS), tumour necrosis factor receptor-associated periodic fever syndrome (TRAPS), Cryopyrin-Associated Periodic Syndrome (CAPS) and 1 pyogenic arthritis, pyoderma gangrenosum and acne syndrome (PAPA).

## Results

Thirty nine patients were identified: 28 SJIAS and 11 AD (7 HIDS, 2 TRAPS, 1 PAPA, 1 CAPS). Sixteen of them had received canakinumab, 35 anakinra and 12 had received both consecutively. Their mean age were 8.7 years (IQR 4.4-13.2).

At least one moderate-severe infection happened in 10/39 (25%) patients. The mean period from the first dose to the first infection was 1.78 years (range 0.3-3.75, IQR 1.05-2.65).

In the SJIA group 3/28 (10%) of the patients had at least one outstanding infection with a total of 7 episodes. In the AD group 5/11 (45%) developed at least one infection, with a total of 10 episodes, all of them in patients with HIDS. The 71% of patients with HIDS had at least one moderate-severe infection. Three patients accumulated the 52% of the episodes.

While receiving anakinra the infection rate was 11,6 episodes x 100 patients/year of treatment and 14, 4 while receiving canakinumab (RR 1.2). Table 1.

**Table 1:** 

	SJIA: 28 patients	AD: 11 patients
Canakinumab	1 pneumonia	1 Herpes Zoster
		1 Soft tissue infection
		1 Recurrent low urinary tract infection

Anakinra	4 pneumonia	1 pneumonia
	1 HPV infection	1 sepsis
	1 severe VEB infection	1 severe herpetic stomatitis
		1 Recurrent low urinary tract infection
		2 oropharingeal candidiasis

## Conclusion

This study suggests a similar incidence of infections in both groups of treatment. Nevertheless we have observed a higher incidence of infections in the AD Group compared to the SJIA group.

## Disclosure of interest

L. Fernandez-Silveira: none declared. M. González-Fernández: none declared. B. López-Montesinos: none declared. S. Benito-Costey: none declared. I. Calvo-Penadés: grant / research support from Colaboration in clinical trials Novartis.

